# Biochemical Characterization and Evaluation of a *Brugia malayi* Small Heat Shock Protein as a Vaccine against Lymphatic Filariasis

**DOI:** 10.1371/journal.pone.0034077

**Published:** 2012-04-05

**Authors:** Gajalakshmi Dakshinamoorthy, Abhilash Kumble Samykutty, Gnanasekar Munirathinam, Gangadhar Bhaurao Shinde, Thomas Nutman, Maryada Venkatarami Reddy, Ramaswamy Kalyanasundaram

**Affiliations:** 1 Department of Biomedical Sciences, University of Illinois College of Medicine at Rockford, Rockford, Illinois, United States of America; 2 Department of Biochemistry, Mahatma Gandhi Institute of Medical Sciences, Sevagram, Maharashtra, India; 3 Department of Biochemistry, Rashtrasant Tukadoji Maharaj Nagpur University, Nagpur, Maharashtra, India; 4 Helminth Immunology Section, National Institutes of Health, Bethesda, Maryland, United States of America; New England Biolabs, United States of America

## Abstract

Filarial nematodes enjoy one of the longest life spans of any human pathogen due to effective immune evasion strategies developed by the parasite. Among the various immune evasion strategies exhibited by the parasite, Interleukin 10 (IL-10) productions and IL-10 mediated immune suppression has significant negative impact on the host immune system. Recently, we identified a small heat shock protein expressed by *Brugia malayi* (BmHsp12.6) that can bind to soluble human IL-10 receptor alpha (IL-10R) and activate IL-10 mediated effects in cell lines. In this study we show that the IL-10R binding region of BmHsp12.6 is localized to its N-terminal region. This region has significant sequence similarity to the receptor binding region of human IL-10. *In vitro* studies confirm that the N-terminal region of BmHsp12.6 (N-BmHsp12.6) has IL-10 like activity and the region containing the alpha crystalline domain and C-terminus of BmHsp12.6 (BmHsp12.6αc) has no IL-10 like activity. However, BmHsp12.6αc contains B cell, T cell and CTL epitopes. Members of the sHSP families are excellent vaccine candidates. Evaluation of sera samples from putatively immune endemic normal (EN) subjects showed IgG1 and IgG3 antibodies against BmHsp12.6αc and these antibodies were involved in the ADCC mediated protection. Subsequent vaccination trials with BmHsp12.6αc in a mouse model using a heterologous prime boost approach showed that 83% protection can be achieved against *B. malayi* L3 challenge. Results presented in this study thus show that the N-BmHsp12.6 subunit of BmHsp12.6 has immunoregulatory function, whereas, the BmHsp12.6αc subunit of BmHsp12.6 has significant vaccine potential.

## Introduction

Lymphatic filariasis caused by the filarial nematodes *Wuchereria bancrofti*, *Brugia malayi*, and *Brugia timori* affects more than 120 million people worldwide [1]. Mass drug administration program by the World Health Organization, significantly reduced the incidence rate of lymphatic filariasis in many parts of the world [2]. However, additional approaches such as use of a vaccine can speed up the effort to eradicate the infection from endemic regions. There are no effective vaccines currently available to control this infection although several candidate vaccine antigens have been reported by several groups including our laboratory [3], [4], [5].

Lymphatic filarial parasites evade the primary line of defense at the skin site and migrate to the lymphatics, where they develop into mature adults and produce microfilariae that are released into the circulation. Individuals with active filarial infection and circulating microfilariae show peripheral blood T-cell hypo responsiveness have poor Th1 type responses and produce high levels of spontaneous IL-10 [6], [7], [8]. This spontaneous production of IL-10 appears to be parasite mediated [9]. In one of our previous studies [10] when we screened a phage display cDNA expression library of *B. malayi* L3 with IL-10R we identified small heat shock protein 12.6 kDa of *B. malayi* (BmHsp12.6) as an IL-10R binding protein. However, we did not identify the peptide sequence of BmHsp12.6 that binds to IL-10R. Nevertheless, our studies showed that rBmHsp12.6 can induce IL-10-like proliferative effects *in vitro* in MC/9 cell lines [10]. In the present study we mapped the IL-10R binding region of BmHsp12.6 and evaluated if this binding region has IL-10 like activity.

The small heat shock proteins (HSP) are a diverse family of 12–43 kDa proteins that assemble into large multimeric structures and functions as chaperone by preventing protein aggregation. Small HSPs contain a conserved α-crystalline domain that is important for its chaperone function [11]. Since BmHsp12.6 also contains α-crystalline domain, in this study we tested if rBmHsp12.6 has chaperone like function. Other reported properties of small HSPs include inhibition of apoptosis [12], actin polymerization and contribution to the optical properties of the eye lens [13]. Transcriptome analysis on *B. malayi* L3 showed that small HSPs are upregulated during the transition of L3 from mosquitoes into mammalian hosts [14]. Thus, when the *B. malayi* pathogen enters the mammalian host, the stress imposed by the host might lead to increased BmHsp12.6 synthesis. Given the chaperone activity of HSPs, these upregulated BmHsp12.6 can protect the parasite proteins from damage. Another potential function of the upregulated BmHsp12.6 could be to modulate host immune responses, as BmHsp12.6 has IL-10 like function. This in turn can suppress the immune response initially and help establish the infection in the host. Recently, Wolbachia HSP60 was identified to contribute to the immune modulation seen in filarial patients [15]. Therefore, BmHsp12.6 appears to be a critical molecule for survival of the parasite in the host. Given its potential immunoregulatory role, BmHsp12.6 is also an attractive target for developing a vaccine against *B. malayi*. HSP-based vaccines have been successfully developed against tuberculosis [16], [17], cancers [18] and against various infectious agents [19]–[21]. Immunization of mice with recombinant HSP60 conferred protection against *Histoplasma capsulatum* [22] and *Porphyromonas gingivalis* [23]. Thus, HSP appears to be a good vaccine candidate. Interestingly, HSP is also shown to be an excellent vehicle for vaccine development [15], [22]. Antigens genetically fused to hsp70 were shown to elicit strong and long-lived humoral and cellular immune responses in the absence of adjuvant [24]. Epitope analysis on the BmHsp12.6 showed that it contains several potential T cell, B cell and CTL epitopes. Therefore, in this study we evaluated the vaccine potential of BmHsp12.6 and its subunits against *B. malayi* in a mouse model.

## Methods

### Ethics Statement

Use of human subjects in this study was approved by the IRB committees at Department of Biochemistry, Mahatma Gandhi Institute of Medical Sciences, Sevagram, India and at the University of Illinois, Rockford, USA. Informed written consent in native language was obtained from all the subjects before collecting the samples.

Humane use of animals was performed in this study according to the guidelines for the care and use of laboratory animals and with the rules formulated under the Animal Welfare Act by the U.S. Department of Agriculture. The protocol was approved by the IACUC Committee of the University of Illinois, College of Medicine at Rockford and performed at a facility accredited by AAALAC and USDA.

### Parasites

*Brugia malayi* L3 were obtained from the NIAID/NIH Filariasis Research Reagent Resource Center (FR3) at the University of Georgia, Athens, GA.

### Human sera Samples

Blood samples were collected after taking informed consent from clinically diagnosed filarial patients and from healthy adult individuals residing in Sevagram and surrounding villages in Maharastra State, which are non-coastal endemic areas for nocturnally periodic *W. bancrofti* infection. Samples were also collected from volunteers who live in areas that are non endemic for filariasis. Parasitological examination of all individuals was done by detection of microfilariae in night blood smears. The presence of mf was further confirmed by membrane (Millipore-5 m filters) filtration of 1.0 ml of heparinized venous blood [25]. The presence of circulating antigen was detected using a WbSXP based enzyme-linked immunosorbent assay (ELISA) [26]. About 10 ml of blood samples (5 ml in heparinized tubes) were collected from the following clinical groups of subjects (a) Endemic normal (EN) subjects, these are individuals who are asymptomatic and non-microfilaraemic residing in Sevagram (b) Asymptomtic microfilaraemic subjects (MF) were classified based on the presence of circulating mf in blood. (c) Chronic Pathology (CP) patients include those subjects who exhibit lymph edema and other chronic clinical symptoms of filariasis. These CP patients did not carry circulating mf and (d) Non-endemic normal (NEN) subjects who live in non-endemic areas and have no circulating parasites or antibodies and show no evidence of any clinical lymphatic filarial disease. These are healthy volunteers from filariasis free regions in India such as Chandigarh, Haryana, Punjab and Kashmir. Sera were separated from these blood samples and were stored at −80°C until use.

### Expression and purification of recombinant *Brugia malayi* heat shock protein

Recombinant *B. malayi* small heat shock protein 12.6 kDa (rBmHsp12.6) was expressed and purified using an immobilized cobalt metal affinity column chromatography as described earlier [10]. Briefly, the full-length gene sequence of *BmHsp12.6* was cloned into pRSET-A vector (with an N-terminal hexahistidine tag) and transformed into BL21(DE3) containing pLysS (Invitrogen, Carlsbad, CA) to minimize toxicity due to the protein. When absorbance of the cultures reached 0.6 OD value, 1 mM of IPTG (isopropyl thio-d-galacto pyranoside) was added to the cultures and incubated for an additional 3 hours to induce gene expression. The histidine tagged recombinant protein was purified using an immobilized cobalt metal affinity column chromatography (Clontech, Mountain View, CA) as per manufacturer's recommendations. Recombinant protein separated in a 15% SDS-PAGE and stained with coomassie brilliant blue R250 showed a single band (data not shown). After purification, rBmHsp12.6 protein was passed through polymyxin B columns (Thermo Fisher, Rockford, IL) to remove any contaminating endotoxin. Levels of endotoxin in the final recombinant protein preparation were confirmed by LAL assay (Gen Script, Piscataway, NJ) and were found to be below 1 IU/ml.

### Structure and motif analysis on BmHsp12.6

Analysis of the secondary structure and protein-protein interaction site of BmHsp12.6 was predicted at PDBsum and at the Predict Protein E-mail server at the European Molecular Biology Laboratory [EMBL], Heidelberg, as described by Rost *et al* [27]. Motif scanning was done using PROSITE pattern analysis to identify the functional motifs in BmHsp12.6.

### Chaperone assay

Chaperones can bind to cellular proteins and protect them from heat damage. When proteins are exposed to heat damage, they aggregate (thermal aggregation) and chaperones can prevent this thermal aggregation. To determine if BmHsp12.6 can prevent thermal aggregation, we used citruline synthase (CS) (Sigma, St. Louis, MO) as a substrate protein to induce thermal aggregation. CS was selected because this protein is a natural cellular protein and is highly sensitive to heat denaturation. Thermal aggregation assay was performed as described previously from our laboratory [28]. Briefly, 1 µM of CS was exposed to 45°C in the presence or absence of BmHsp12.6 (2 µM) suspended in 50 mM of sodium phosphate pH 7.4 buffer containing 100 mM NaCl. BSA was used as a control. CS was incubated with BmHsp12.6 at a molar ratio of 1∶2 for various time intervals from 0 to 40 minutes. Thermal denaturation (aggregation) was monitored spectrophotometrically at 300 nm.

### *In vitro* peptide binding assay for chaperone activity

Chaperons can bind to denatured proteins. To test if BmHsp12.6 can also bind to denatured proteins, we chemically denatured CS and another protein, luciferase (LUC) with 6 M guanidine hydrochloride overnight at 4°C as described previously [28]. Native and chemically denatured proteins were then coated on to 96 well plates overnight at 4°C. After washing with PBS, wells were blocked with 3% BSA at room temperature. Following further washing, wells were incubated with his tag rBmHsp12.6 or control filarial protein (his tag abundant larval transcript-2) for 1 hr at 37°C. After washing with PBS, optimally diluted anti-his tag HRP conjugate was added and incubated at 37°C for 1 hr. After final washing, color was developed with OPD (o-phenylenediamine dihydrochloride) substrate and OD was measured at 450 nm.

### Epitope mapping on BmHsp12.6

B-cell epitopes, T- cell epitopes and CTL epitopes on BmHsp12.6 sequences were predicted using Immune Epitope Database and Analysis Resource (IEDB) at http://epitope2.immuneepitope.org/home.do. Propred [29] tool was also used for prediction of HLA binding peptides, to identify putative T epitopes.

### Peptide synthesis

Two peptide fragments of BmHsp12.6; N-BmHsp12.6 (aa 1–36) and BmHsp12.6αc (aa 61–113) were synthesized chemically at Genescript Corporation.

### Assay to determine IL-10-like function

BmHsp12.6 binds to huIL-10R and induces IL-10-like function [10]. To determine if the huIL-10R binding peptide of BmHsp12.6 is involved in the IL-10-like function, we used an IL-10 dependent MC/9 cell proliferative assay as described previously [10]. Briefly, the mast cell line, MC/9 (ATCC, Manassas, VA) was maintained in DMEM supplemented with 10% FBS, 0.05 mM 2-ME, and 10% Rat T-STIM (Becton Dickenson, San Jose, CA) at 37°C in 5% CO_2_ environment. About 5×10^3^ MC/9 mast cells were suspended in DMEM containing IL-3 (10 ng/ml), IL-4 (10 ng/ml) and polymyxin B (10 µg/ml) and plated on a flat bottomed 96 well tissue culture plates and stimulated with 10 µg/ml of N-BmHsp12.6 peptide or BmHsp12.6αc peptide or rBmHsp12.6 protein. Recombinant huIL-10 (100 ng/ml) was used as a positive control. In some wells polyclonal antibodies against N-BmHsp12.6 or BmHsp12.6αc peptides (50 µl) was added along with the peptides or proteins to block the function of respective proteins. Sera from control Balb/c mice was used as a negative control in the inhibition studies. After 72 h of culture, cell proliferation was determined using a cell proliferation kit purchased from Dojindo Molecular Technologies Inc., Gaithersburg, MA.

### Anti-BmHsp12.6 antibody levels in human sera

Sera samples from 20 MF, CP, EN or NEN subjects were analyzed for the presence and titer of IgG antibodies against rBmHsp12.6 and its peptides using an indirect ELISA as described previously [5]. Briefly, wells of a 96 well microtitre plate were coated with rBmHsp12.6 (1 µg/ml), N-BmHsp12.6 peptide (5 µg/ml) or BmHsp12.6αc peptide (5 µg/ml) in carbonate buffer, pH 9.6 overnight at 4°C and blocked with 3% BSA for 1 h at 37°C. Sera samples (1∶100 diluted) were added to the wells and the plates were incubated overnight at 4°C. After washing the wells, HRP labeled mouse anti-human IgG was added (1∶5000) and incubated further for 1 hr at 37°C. Color was developed using OPD (o-phenylenediamine dihydrochloride) substrate. Absorbance was measured at 450 nm in a microplate reader (Bio-Rad, Hercules, CA). We also determined the isotype of IgG antibodies against rBmHsp12.6 and its peptides (N-BmHsp12.6 and BmHsp12.6αc) in the sera of subjects using an isotype specific ELISA. Biotinylated mouse monoclonal anti-human IgG1, IgG2, IgG3 and IgG4 were used as the secondary antibodies and color was developed with avidin-HRP conjugate (Sigma) as the secondary antibodies.

### Antigen-specific proliferation of human peripheral blood mononuclear cells

Peripheral blood mononuclear cells (PBMC) were isolated from the heparinized venous blood of study subjects using histopaque 1077 column (Sigma). Viability of the cells was determined by tryphan blue dye exclusion method. PBMC were then cultured in 96 well tissue culture plates at a concentration of 2×10^5^ cells/well in RPMI 1640 supplemented with 10% FCS. PBMC were stimulated either with rBmHsp12.6 antigen or non-specific recombinant protein (rSmGBF) or ConA (1 µg/ml) or with medium alone (unstimulated) in triplicate wells. PBMC were stimulated in triplicate wells and the plates were incubated at 37°C in 5% CO_2_. 72 h after incubation, cell proliferation was measured by an MTT assay (Cell Titer 96 R aqueous non-radioactive cell proliferation assay, Promega, Madison, WI). Stimulation index was calculated and the results were expressed as Mean S.I ± S.D.

### Cytokine assay

Levels of Interferon-γ (IFN-γ), Interleukin-4 (IL-4) and Interleukin-10 (IL-10) in the culture supernatants of PBMC stimulated above with rBmHsp12.6 were estimated using Cytokine ELISA kits (E-biosciences, San Diego, CA) as per the manufacturer's instruction. Concentration of each cytokine in the culture supernatant was plotted from a standard curve using recombinant IFN-γ, rIL-4 or rIL-10 and the data was expressed as pg/ml. Cytokine values in the culture supernatant of control cells incubated with rSmGBF or media alone was used as the background value.

### Cloning of Codon optimized BmHsp12.6 into pVAX vector for DNA vaccine

Codon optimized *BmHsp12.6* genes encoding N-BmHsp12.6, BmHsp12.6αc or full length BmHsp12.6 protein were cloned into eukaryotic expression vector pVAX (Invitrogen, Carlsbad, CA) using insert specific primers (forward primer: for *N-BmHsp12.6*; 5′-CGCGGATCCATGGAG GAGAAGGTGG-3′, for *BmHsp12.6αc*; 5′-CGCGGATCCATGGTCATTCACTGCAGG-3′ and for *BmHsp12.6*; 5′-CGCGGATCCATGGAAGAGAAGGTGGTG-3′) containing BamHI site and (reverse primer: for *N-BmHsp12.6*; 5′-CCGGAATTCTCAGGCTTTCTTCTTGGC-3′, for *BmHsp12.6αc*; 5′-CCGGAATTCTCACTTGGCAGCGATGA-3′ and *BmHsp12.6*; 5′-CCGGAA TT CTCACTTGTCGTTGGTG-3′) containing EcoRI site. PCR parameters were as follows: 94°C of denaturation for 30 s, 50°C of primer annealing for 30 s, 72°C of primer extension for 30 s for 30 cycles; a final extension of 5 min was performed at 72°C. Insert DNA was sequenced to ensure authenticity of the cloned nucleotide sequence on both strands. Plasmids were maintained and propagated in *E. coli* Top10F′ cells. Subsequently plasmids were purified using endotoxin free plasmid extraction kit (Qiagen, Hilden, Germany). DNA was analyzed by agarose gel electrophoresis and quantified by spectrophotometry (OD 260/280, ratio>1.8).

### Immunization of mice

Six-weeks old male Balb/c mice purchased from Charles River Laboratories were used in these experiments and divided into 8 groups. Each group consisted of five (5) mice and all mice were immunized intraperitonealy (i.p). For DNA immunization, mice were injected with four doses of 100 µg of pVAX *N-BmHsp12.6 DNA* or pVAX *BmHsp12.6αc DNA* or pVAX *BmHsp12.6 DNA*. For recombinant protein immunization mice were injected with four doses of 15 µg of rBmHsp12.6. For prime boost immunization, mice were primed twice with 100 µg of pVAX *N-BmHsp12.6 DNA* or pVAX *BmHsp12.6αc DNA* or pVAX *BmHsp12.6* DNA and followed by two booster doses of 15 µg of N-BmHsp12.6 peptide or BmHsp12.6αc peptide or rBmHsp12.6 protein respectively. Alum was used as an adjuvant for rBmHsp12.6. However, synthetic peptide vaccines, despite being pure antigens, tend to be poorly immunogenic when administered with alum [30]. Therefore, saponin (0.2% w/v) was used as an adjuvant for N-BmHsp12.6 and BmHsp12.6αc peptides. Saponin has been used as an adjuvant with a variety of antigens, because it can enhance immune responses to subunit vaccines in laboratory animals [31] and humans [32]. Control animals received 100 µg of pVAX vector alone and/or equal volumes of respective adjuvants at the same interval. Blood samples were collected from each mouse before immunization and one month after the last booster dose and sera separated. Pooled sera of immunized mice containing antibodies against rBmHsp12.6, N-BmHsp12.6 peptide and BmHsp12.6αc peptide was used in neutralization assays.

### Anti-BmHsp12.6 antibody levels in the sera of mice

Levels of anti-BmHsp12.6 IgG antibody in the sera (1∶100) were determined using an indirect ELISA as described previously [5]. IgG1, IgG2a, IgG2b and IgG3 anti-BmHsp12.6 antibody levels were also determined using a mouse antibody isotyping ELISA kit (ThermoFisher Scientific). Color was developed with OPD chromogen substrate and the absorbance was measured at 450 nm in an ELISA reader (BioRad). Sera that showed high titer of IgG antibodies were used in the Antibody Dependent Cellular Cytotoxicity assay described below.

### Antibody-dependant cellular cytotoxicity (ADCC) assay

*In vitro* ADCC assay was performed as described previously [33], [5]. Briefly, ten (10) L3 of *B. malayi* were incubated with 2×10^5^ peritoneal cells (PEC) collected from normal mice, 50 µl of pooled mouse sera samples and 50 µl of RPMI 1640 media in a 96 well culture plate (Thermo Fisher Scientific). After 48 h of incubation at 37°C and 5% CO_2_, larval viability was determined at 400× using a light microscope. Larvae that were limpid and damage were counted as dead. In addition, dead larvae also had clumps of cells adhered to it and were more transparent than live ones. Larvae that were active coiled and translucent were counted as live. ADCC was estimated as the percent larval death calculated using the formula:
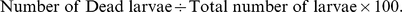



ADCC assay was also performed with pooled human sera samples as described above except that the human sera samples were incubated with 2×10^5^ PBMC collected from normal healthy subjects and 6–12 *B. malayi* L3 for 48 h at 37°C and 5% CO_2_. Larval viability and death was determined as described above.

### Depletion of anti-BmHsp12.6 antibodies from human sera

BmHsp12.6 specific antibodies were depleted from the pooled sera of EN subjects by passing through cobalt IMAC resin column coupled with his-tagged rBmHsp12.6 as described by Veerapathran et al. [5]. Briefly, 1 mg of his-tagged rBmHsp12.6 was coupled to 2 ml bed volume of IMAC resin for 2 hrs at 37°C. After washing the resin once with 10 ml of PBS (pH.8), 200 µl of pooled sera was added and incubated overnight at 4°C. After incubation, the resin mixture was centrifuged for 2 min at 750 rpm and the supernatant was collected. Depletion of anti-BmHsp12.6 antibodies in the supernatant was confirmed by an ELISA as described above (Data not shown). Anti-BmHsp12.6 antibodies depleted sera sample was then used in an ADCC assay.

### Evaluation of vaccine-induced protection in mice

Vaccine potential of BmHsp12.6 or its peptides were evaluated using a mouse model of challenge infection. Mice were immunized as described above and one month after the last immunization, a micropore chamber containing challenge L3 was implanted into the peritoneal cavity of mice as described by Abraham et al. [34]. Briefly, micropore chambers were assembled using 14×2 mm plexi rings (Millipore Corporations, Bedford, MA) and 5.0 µm nucleopore polycarbonate membranes (Millipore Corporations). The membranes were attached to the plexi glass rings with cynoacrylic adhesive and dental cement. The chambers were immersed overnight at 37°C in sterile RPMI medium containing gentamycin and antimycotic solution. Before the challenge experiments, 20 live infective L3 suspended in RPMI1640 medium supplemented with 10% heat inactivated fetal bovine serum (FBS), were introduced into the micropore chambers and the opening was sealed with dental cement. Micropore chamber containing L3 were then surgically implanted into the peritoneal cavity of each mouse under anesthesia. Aseptic conditions were followed for the surgical procedures. After 48 h of implantation, animals were sacrificed and the chambers were recovered from the peritoneal cavity. Contents of each chamber were emptied and larvae were examined microscopically for adherence of cells and for larval death. Dead and live larvae were identified as described above under ADCC. The percentage of larval death was expressed as the number of dead parasites/number of total parasites recovered×100.

### Splenocytes proliferation assay

Spleens were collected from all mice from the above experiment and single cell suspension of spleen cells was prepared. Approximately 2×10^5^ cells/well suspended in complete RPMI1640 medium supplemented with 10% heat inactivated FBS were incubated at 37°C and 5% CO_2_ for 72 hrs with either 1 µg/ml of antigens (rBmHsp12.6, N-BmHsp12.6 peptides or BmHsp12.6αc peptides) or non filarial recombinant protein (rGBF) or ConA or with medium alone. After incubation, cell proliferation was determined using cell counting kit (CCK-8) purchased from Dojindo Molecular Technologies, Inc, Gaithersburg. Stimulation index of spleen cell proliferation was calculated using the formula: Absorbance of stimulated cells÷Absorbance of unstimulated cells.

### Statistical Analysis

Statistical analysis was performed using GraphPad prism software version 5. Comparisons between two individual data points were made using Student's t-test. For multiple comparisons, non-parametric Kruskal–Wallis test was used. Statistical significance level was set at p<0.05.

## Results

### BmHsp12.6 has an alpha-crystalline domain

**Figure 1:**
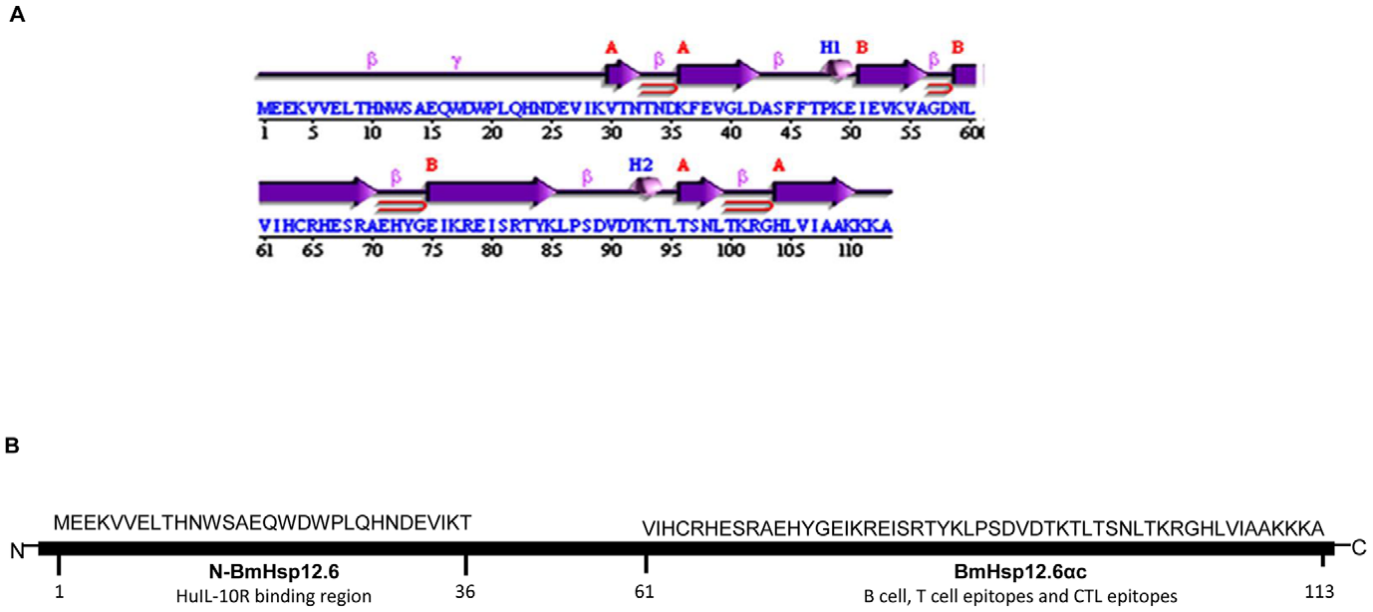
Structural analysis of BmHsp12.6. (**A**) Secondary structure prediction of BmHsp12.6 showing α-helices (H1 and H2), strands by their sheets A, B, (**β**)- beta turn, (**γ**)- gamma turn and beta hairpin (**⊃**). (**B**) Graphical representation showing N-BmHsp12.6 and BmHsp12.6αc peptide sequences and their functional roles in the BmHsp12.6 protein.

Secondary structure prediction analysis of BmHsp12.6 protein showed the presence of an alpha-crystalline domain that has an immunoglobulin core consisting of seven β-strands arranged in two anti-parallel sheets (Figure 1A). The secondary structure prediction also showed that the N- terminus fragment (aa 1–30) has no predicted secondary structure.

### HuIL-10R binding region of BmHsp12.6 resides within the N-BmHsp12.6 sequences

Previous studies showed that BmHsp12.6 binds to human IL-10 receptor I α chain [19]. To identify the IL-10 receptor binding site on BmHsp12.6, we performed a predictive protein-protein interaction analysis as described previously [35]. Results from the prediction analysis showed that the N-BmHsp12.6 (amino acids from Met^1^ to Asn^26^) has a strong protein-protein interaction region. Further sequence analysis of this region showed that the amino acid sequence from Val^5^ to Glu^42^ has 16% sequence identity and 38% similarity to human IL-10R binding region of human IL-10. These findings suggest that the N-BmHsp12.6 may be involved in the binding of BmHsp12.6 to human IL-10 receptor I α chain.

### BmHsp12.6 protein has similar motifs as human IL-10

Motif analysis performed at the PROSITE showed several putative post-translation modification sites such as N-glycosylation sites (aa 11 to 14 and 98 to 101), protein kinase-c phosphorylation sites (aa 83 to 85 and 100 to 102), casein kinase II phosphorylation sites (aa 68 to 71 and 88 to 91) and N-myristylation sites (aa 40 to 45) on BmHsp12.6. Similar motifs were also observed in human IL-10 further confirming the previous findings that BmHsp12.6 may mimic human IL-10 function [10].

### N-BmHsp12.6 contains IL-10R binding site and BmHsp12.6αc has immunogenic epitopes

Analysis of the BmHsp12.6 sequences showed that the sequences from aa 1–36 had IL-10R binding region. This region was termed N-BmHsp12.6 peptide as this resides in the N-terminus region of BmHsp12.6. Epitope analysis showed that sequences from aa 61–113 contained linear B epitopes, T epitopes and CTL epitopes (Table 1). This region was termed BmHsp12.6αc peptide and is graphically represented in Figure 1B.

**Table 1:** Predicted B-cell, T-cell and CTL epitopes in BmHsp12.6 sequences.

Epitopes predicted^a^	Position of epitope in the aa sequence	Peptide sequence
B-cell epitopes	67–74	SRAEHYGE
	87–101	PSDVDTKTLTSNLT
T-cell epitopes	50–57	IEVKVAGD
	52–60	VKVAGDNLV
	84–92	KLPSDVDTK
	92–100	KTLTSNLTK
	90–98	DTKTLTSNL
	100–108	KRGHLVIAA
	73–81	GEIKREISR
CTL epitopes	78–86	REISRTYKL
	74–82	GEIKREISR
	70–78	AEHYGEIKR

a Immune Epitope Database and Analysis Resource (IEDB) was used for the prediction.

### N-BmHsp12.6 promotes MC/9 mast cell proliferation

**Figure 2:**
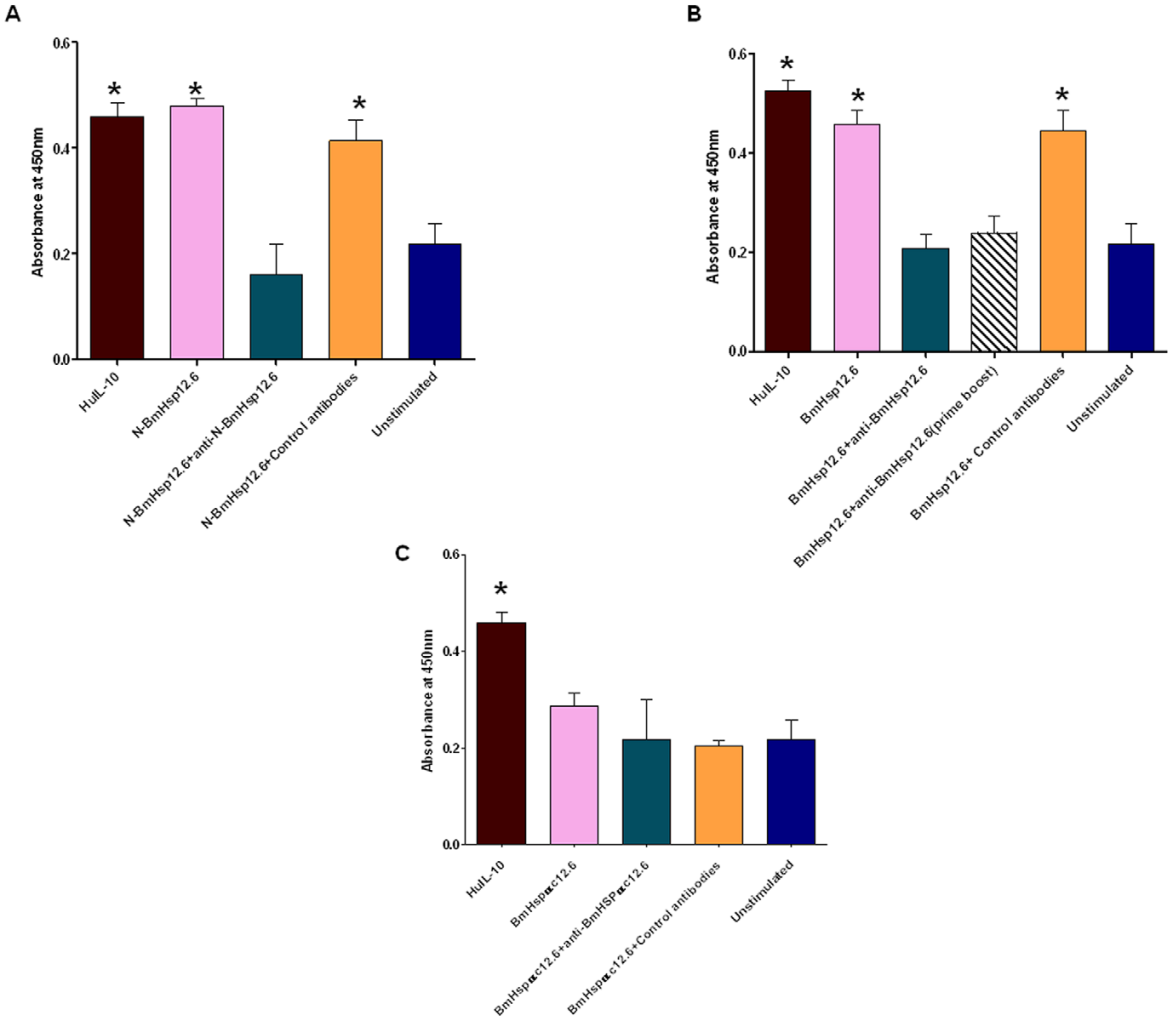
MC/9 proliferation assay. N-BmHsp12.6 peptide enhances the growth of MC/9 mast cells *in vitro*. The effects of different peptides of BmHsp12.6 on MC/9 cells were determined by a cell viability assay. 5×10^3^ cells/ml of MC/9 were stimulated with 10 µg/ml of **A**) N- BmHsp12.6 **B**) BmHsp12.6 and **C**) BmHsp12.6αc (10 µg/ml) for 72 h at 37°C. Sera containing antibodies against the respective proteins were added to certain cultures. Sera samples from normal Balb/c mice served as negative controls. Cells stimulated with rhuIL-10 (100 ng/ml) served as positive control. Data presented is representative of one of three similar experiments. * Significant (p<0.005) compared to all the other groups.

*In vitro* function studies using MC/9 cells showed that when MC/9 cells were incubated with N-BmHsp12.6 peptide (Figure 2A) they proliferated nearly at the same level as with rBmHsp12.6 or rhuIL-10 (Figure 2B). The MC/9 growth stimulating activity of N-BmHsp12.6 peptide or rBmHsp12.6 were abolished when pooled mouse sera containing polyclonal antibodies against N-BmHsp12.6 peptide or rBmHsp12.6 were added to the cultures suggesting that the effect of N-BmHsp12.6 peptides and rBmHsp12.6 on MC/9 is specific. No inhibitory effect was observed when control sera were used with N-BmHsp12.6 peptide or rBmHsp12.6. Similarly, MC/9 cells cultured in the presence of BmHsp12.6αc peptide showed no significant proliferation compared to controls (Figure 2C). These results suggest that the IL-10R binding N-BmHsp12.6 also has IL-10-like function *in vitro* on MC/9 cells.

### BmHsp12.6 is a chaperone

**Figure 3:**
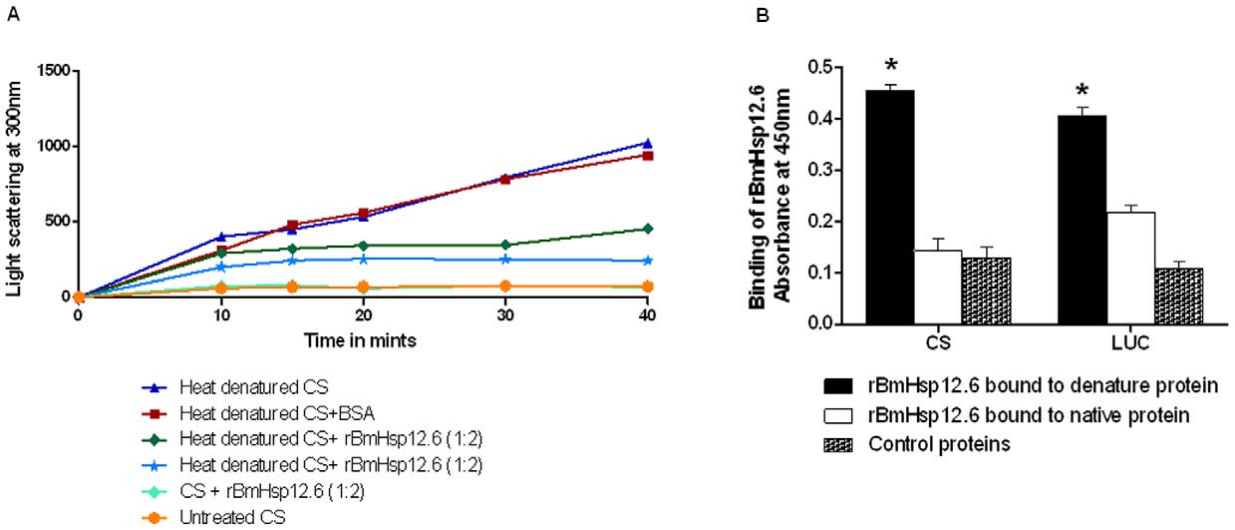
Recombinant BmHsp12.6 prevents thermal aggregation of proteins. (**A**) Citrulline synthase (CS) were heat denatured at 45°C in the presence and absence of rBmHsp12.6 at different time intervals (0–40 min). BSA served as control. Thermal aggregation of proteins was determined spectrophotometrically by measuring the light scatter at 300 nm. Results show that ratio of CS: BmHsp12.6 (1∶2) was sufficient to prevent thermal aggregation of CS. Data presented are representative of three similar experiments. (**B**). **BmHsp12.6 can bind to denatured proteins**. Binding of rBmHsp12.6 to denatured Citruline synthase (CS) and luciferase (LUC) was determined using an ELISA. CS or luciferase was denatured with 6 M guanidium hydrochloride overnight at 4°C. Wells of 96 wells plate was then coated with the denatured or native CS or LUC and binding of his-tag rBmHsp12.6 or control filarial protein to the coated proteins (CS and LUC) was then analyzed using HRP-labeled penta- his antibodies by ELISA. Results show that BmHsp12.6 preferentially binds to denatured proteins. * Significant (p<0.005) binding of BmHsp12.6 to denatured proteins compared to control and native proteins.

Majority of the heat shock proteins reported to date have chaperone function. To test if BmHsp12.6 also has similar chaperone function, we performed a thermal aggregation reaction using Citrulline synthase (CS) as a substrate. Incubation of CS at 45°C resulted in unfolding of the protein and subsequent aggregation within 10 minutes (Figure 3A). Addition of rBmHsp12.6 to CS protein (at a molar ratio of 1∶2) before the heat treatment significantly (P<0.01) inhibited the thermal aggregation of CS protein (Figure 3). Addition of a non-chaperone control protein, BSA to CS had no effect on the heat-induced aggregation of CS protein.

Another key function of chaperone proteins is that they can specifically bind to denatured proteins. To test if BmHsp12.6 can specifically bind to denatured proteins, we incubated rBmHsp12.6 with CS protein or luciferase (native and denatured) proteins. These studies showed that rBmHsp12.6 preferentially binds to denatured protein compared to native or control proteins (Figure 3B). These findings further confirmed that BmHsp12.6 can act as a molecular chaperone potentially protecting the parasite cellular proteins from the damaging effects of the host.

### EN subjects carry high levels of circulating anti-BmHsp12.6 antibodies

**Figure 4:**
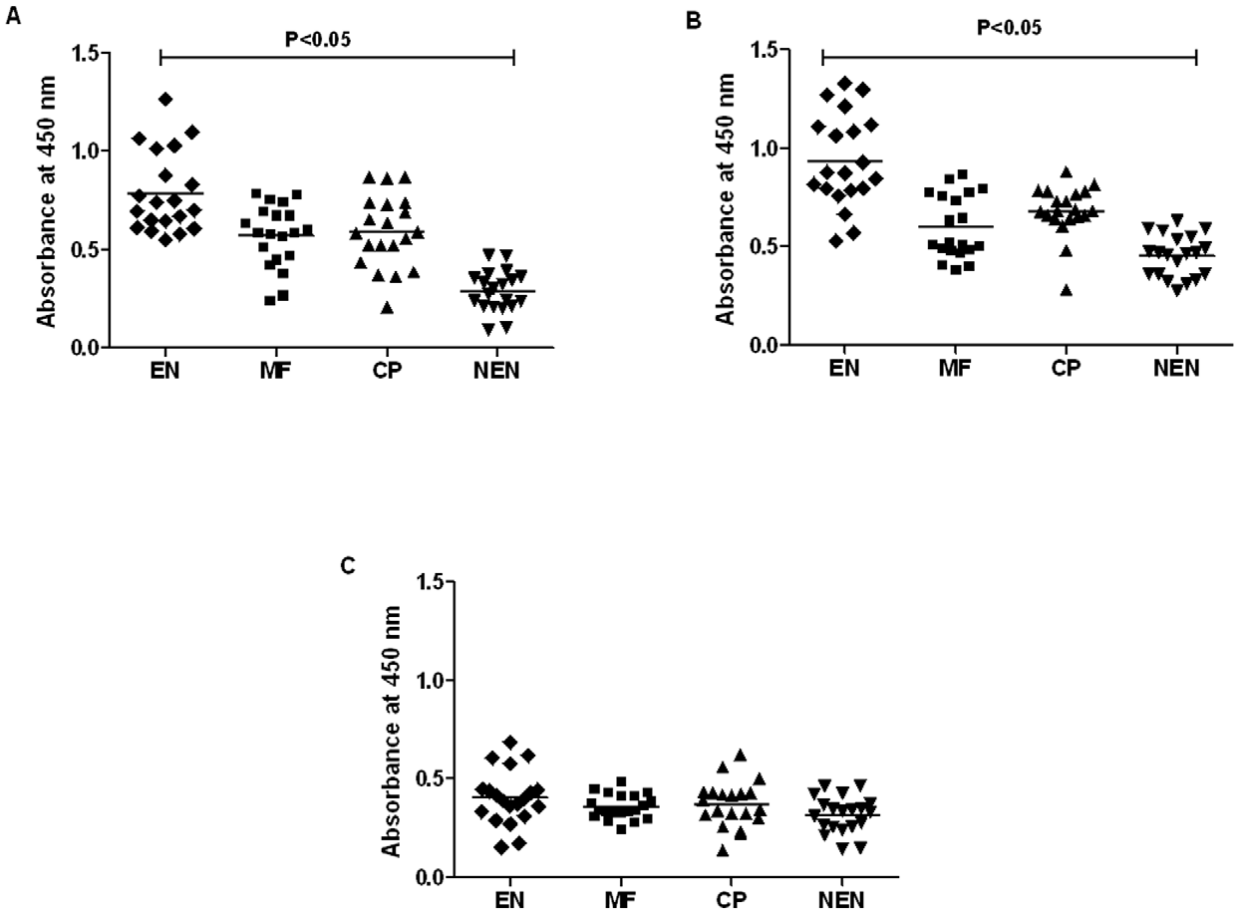
Anti-rBmHsp12.6 IgG antibody levels in the sera of human. Levels of total IgG antibodies against (**A**) rBmHsp12.6 protein, (**B**) BmHsp12.6αc peptide or (**C**) N-BmHsp12.6 peptide in the sera of EN, MF, CP and NEN subjects were measured using an indirect ELISA. A total of 20 sera samples were evaluated from EN, MF, and CP and 10 samples from NEN. Each data point represents sera sample from a single individual. Horizontal lines represent geometric mean value. Data is represented as scatter plot where each dot represents absorbance of individual sera.

**Figure 5:**
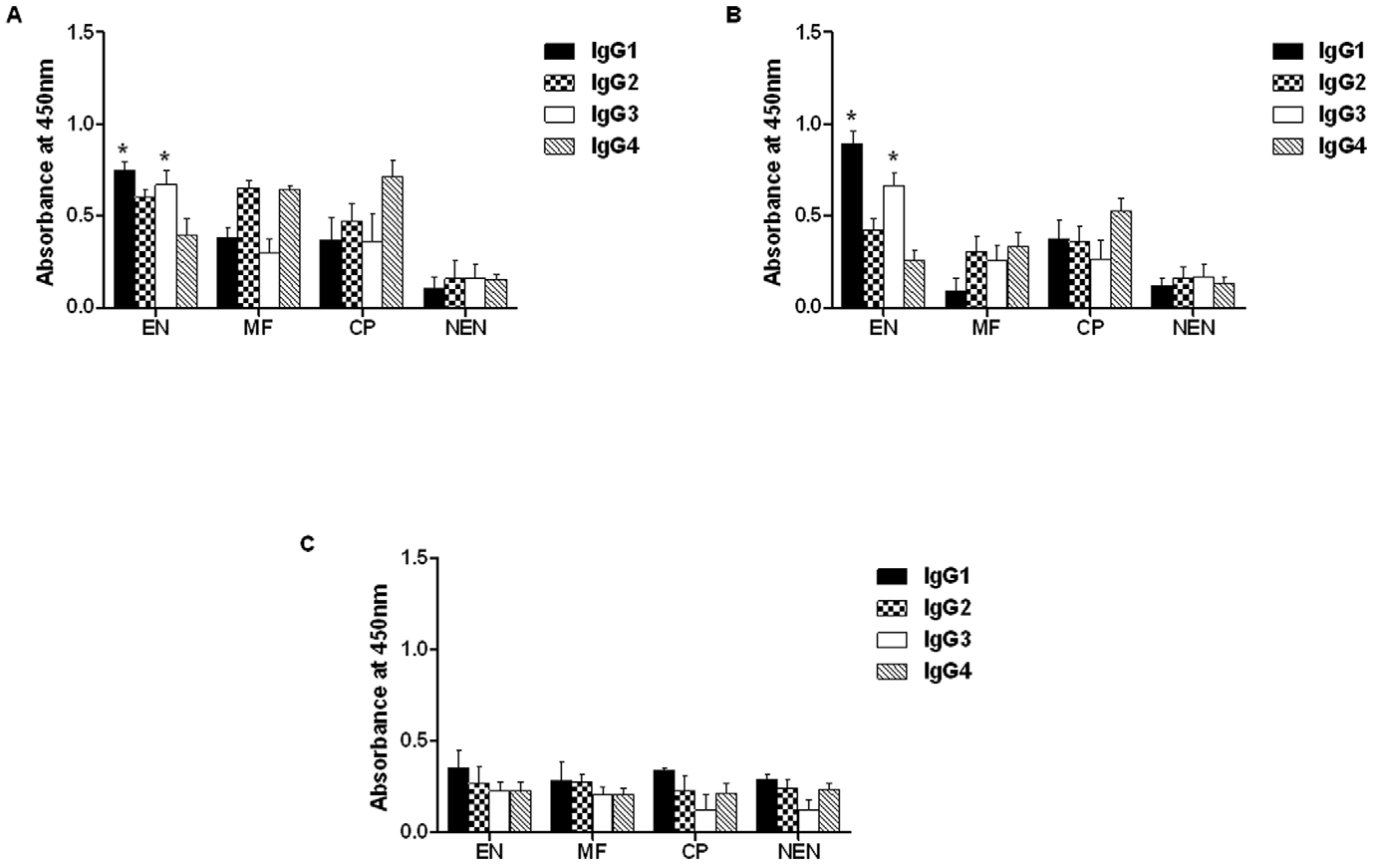
Anti-rBmHsp12.6 IgG antibody isotypes in the human sera. IgG isotype antibodies against (**A**) rBmHsp12.6 protein, (**B**) BmHsp12.6αc peptide or (**C**) N-BmHsp12.6 in the sera of EN, MF, CP and NEN subjects were measured using an indirect ELISA. A total of 20 sera samples were evaluated from EN, MF, and CP from NEN. Each data point represents sera sample from a single individual. Each bar represents the mean ± SD of five patients from each group. * Significant (p<0.05) compared to all the other three groups (CP, MF and NEN).

Epitope mapping on BmHsp12.6 revealed the presence of B-cell, T-cell and CTL epitope regions in the protein (Table 1) suggesting that BmHsp12.6 is potentially a highly immunogenic peptide. Therefore, we assumed that filariasis infected individuals might carry antibodies to BmHsp12.6. Analysis of the titer of IgG antibodies against BmHsp12.6 and its subunits in the sera of EN, CP, MF and NEN subjects showed that the EN subjects had the highest levels of anti-BmHsp12.6 (Figure 4A) or anti BmHsp12.6αc (Figure 4B) antibodies (p<0.001) in their sera. EN subjects did not carry any IgG antibodies against the N-BmHsp12.6 peptides (Figure 4C). Subsequent isotype analysis of the IgG anti-BmHsp12.6 or anti BmHsp12.6αc antibodies showed that IgG1 and IgG3 anti-BmHsp12.6 antibodies were the most predominant antibodies in the sera of EN subjects (Figure 5). Anti-BmHsp12.6 antibodies in the sera of MF carriers were mainly of IgG2 and IgG4 isotype, whereas, anti-BmHsp12.6 antibodies in the sera of CP individuals were predominantly IgG4 isotype. Levels of both IgG1 and IgG3 isotype of anti-BmHsp12.6 antibodies were low or near background level in the sera of MF and CP individuals. Anti-BmHsp12.6αc antibodies in the sera of CP patients carried predominantly IgG4 isotype antibodies. Levels of IgG1, IgG2 and IgG3 isotype of anti-BmHsp12.6αc antibodies were very low in MF individuals. No anti-BmHsp12.6 or anti-BmHsp12.6αc antibodies could be detected in the sera of NEN subjects.

### Anti-BmHsp12.6 antibodies in the sera of EN subjects are involved in ADCC mediated protection

Above results show that antibodies to BmHsp12.6 were present in the sera of infected groups of individuals (MF and CP) and in putatively immune EN subjects. To test if these antibodies are functional, we performed an *in vitro* antibody dependent cellular cytotoxicity assay, where the respective serum (antibodies) is combined with PBMC from a healthy donor and ability of the antibodies to participate in larval killing is evaluated. Results of the ADCC assay show that pooled sera from EN subjects (N = 10) promoted adherence of PBMC to the surface of L3 and caused significant (P<0.05) death of *B. malayi* L3 *in vitro* (Table 2), whereas, pooled sera from MF, CP or NEN failed to induce cell adherence or larval killing (Table 2). To further determine the role of anti-BmHsp12.6 antibodies in this larval killing, we depleted anti-BmHsp12.6 antibodies from the pooled sera of EN subjects and used it in the ADCC assay above. Results show that depletion of anti-BmHsp12.6 antibodies from the sera samples significantly (P<0.05) impaired their ability to participate in the killing of L3 (36%) suggesting that the anti-BmHsp12.6 IgG1 and IgG3 antibodies in the sera of EN subjects may have a role in the killing of L3.

**Table 2:** Antibody-dependant cellular cytotoxicity (ADCC) against *B. malayi* L3 using human serum.

Groups	% Larval death^a^ (Mean ± SD)
Endemic normal (EN) sera	**75±11.85** *
EN sera depleted of anti rBmHsp12.6 antibodies	36±13.82
Non endemic normal (NEN) sera	14±5.90
Microfilaremic (MF) sera	6±5.5
Chronic pathology (CP) sera	9±8.4

a 50 µl of pooled EN, NEN, MF or CP human sera (n = 10) samples were incubated with 2×10^5^ normal PBMC and *B. malayi* L3 at 37°C for 48 hrs. Following incubation, larval viability was determined and percent larval death was calculated. Anti-BmHsp12.6 antibodies were depleted from the EN pooled sera by passing through an affinity column containing immobilized BmHsp12.6. Depleted sera samples are then used in ADCC assay. Values represent mean ± SD of three wells.

* Significant larval death (P<0.005) compared to NEN, MF and CP sera samples.

### BmHsp12.6-specific cells are present in EN subjects

**Figure 6:**
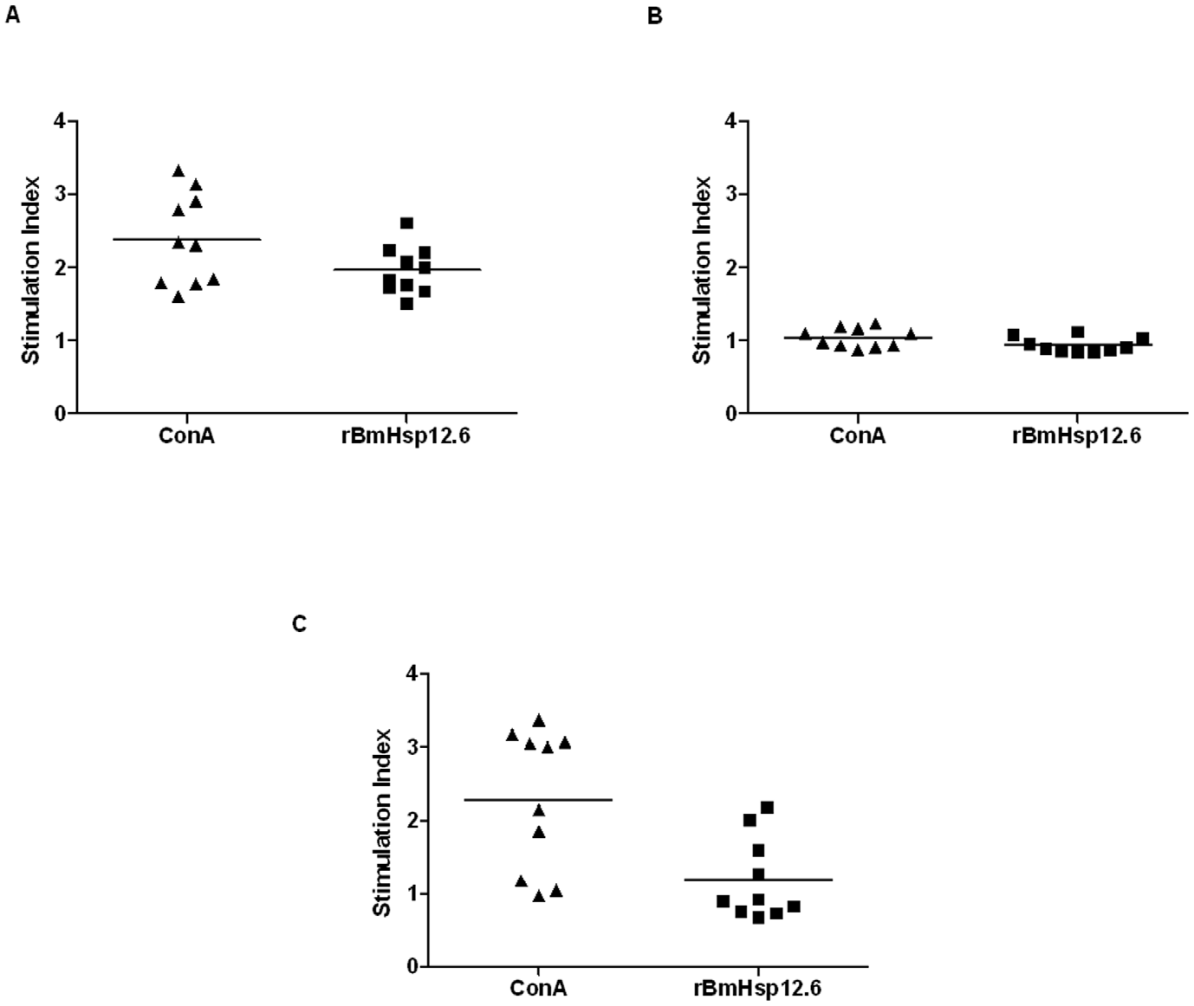
Proliferation of human PBMC stimulated with rBmHsp12.6. PBMC proliferation of (**A**) Endemic Normal (EN), (**B**) Microfilaraemic (MF) and (**C**) Chronic Pathology (CP) groups stimulated with rBmHsp12.6. Concanavalin A (ConA) was used as a positive control. Each data point represents stimulation index (S.I) of individual human sample (n = 10).

Freshly isolated PBMC from different filarial groups (EN, MF and CP, n = 10) were stimulated with rBmHsp12.6 or ConA and proliferative response was measured using an MTT assay. Figure 6 show that PBMC from EN individuals stimulated with rBmHsp12.6 (S.I. 1.6–2.6) showed significant proliferative responses (p<0.05) compared to PBMC from CP individuals (S.I. 0.8–2.1) and MF individuals (S.I. 0.8–1.0). Control wells stimulated with ConA showed S.I. 1.8–3.7 for EN; S.I. 0.9–3.8 for CP and S.I. 0.7–3.1 for MF.

### BmHsp12.6 specific cells in EN subjects predominantly secrete IFN-γ

**Figure 7:**
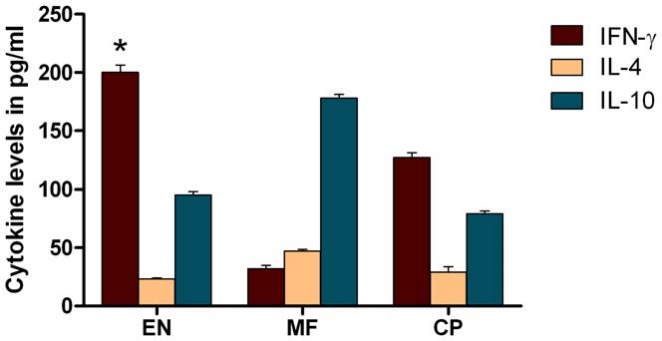
Cytokine levels in human PBMC. Cytokines (pg/ml) in the culture supernatants of human PBMC were measured using an ELISA. Results show that significant level of IFN-γ is secreted by PBMC of EN individuals in response to rBmHsp12.6. Experiments were repeated two times. Each bar represents mean concentration ± S.D. * Significant (p<0.05) IFN-γ secretions compared to other two groups (CP and MF).

Levels of IFN-γ, IL-4 and IL-10 were estimated in the cultures supernatants of PBMC stimulated with rBmHsp12.6. Results showed that rBmHsp12.6 induced secretion of significant levels (193.7 pg/ml) of IFN-γ (p<0.001) from the PBMC of EN subjects compared to PBMC from MF and CP individuals (**Figure 7**). Levels of IL-4 and IL-10 were also slightly increased (27.2 and 98.4 pg/ml respectively) in the culture supernatant of PBMC from EN subjects stimulated with rBmHsp12.6. PBMC from MF subjects secreted significant amounts of IL-10 in response to rBmHsp12.6 and PBMC from CP individuals showed slight increases in IFN-γ but not as significant as the EN subjects. There were no detectable levels of cytokine in the culture supernatants of control PBMC from EN subjects incubated in the media (data not shown).

### BmHsp12.6 and BmHsp12.6αc is a potential vaccine candidate

**Figure 8:**
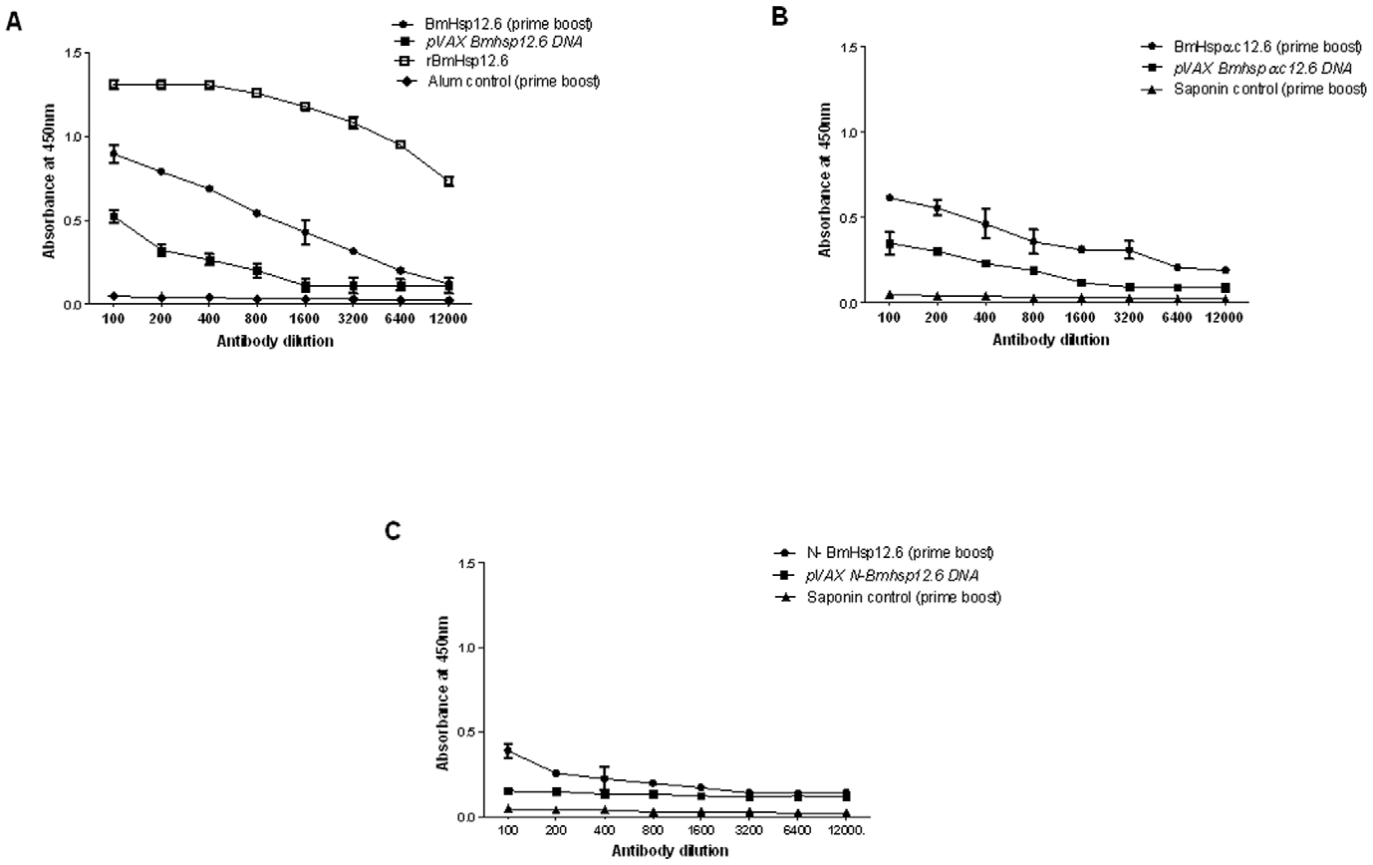
Titer of IgG antibodies. Mice were immunized with **A**) BmHsp12.6, **B**) BmHsp12.6αc or **C**) N-BmHsp12.6 using homologous DNA vaccine regimen or a heterologous prime boost approach. Approximately, 100 ng of peptides or proteins respectively (100 ng/100 µl/well) were coated on to the wells of an ELISA plate and bound serum IgG was detected using an HRP-labeled anti-mouse IgG secondary antibody. Each data point indicates mean (± S.D) value from five animals.

Above results show that putatively immune EN subjects carry antibodies against rBmHsp12.6 is involved ADCC mediated protection. Therefore, to test if rBmHsp12.6 or its peptides (N-BmHsp12.6 and BmHsp12.6αc) has any vaccine potential; we performed a proof-of-concept vaccine trial in Balb/c mice using a DNA, or DNA prime+protein boost immunization regimen. We also immunized one group of mice with rBmHsp12.6 protein. These studies showed that all immunized mice developed significant levels of anti-BmHsp12.6 IgG antibodies (Figure 8). Overall, rBmHsp12.6 protein vaccination gave the highest titer of IgG antibodies (p<0.05) compared to DNA vaccination (Figure 8A). The DNA prime protein boost vaccination regimen also elicited a strong IgG response however the titer was significantly lower than the protein alone vaccination group. Titer of IgG antibodies after prime boost vaccination with BmHsp12.6αc peptide was comparable to those after vaccination with rBmHsp12.6 (Figure 8B). N-BmHsp12.6 peptide was a poor immunogen either as a DNA vaccine or with prime boost vaccination (Figure 8C).

**Figure 9:**
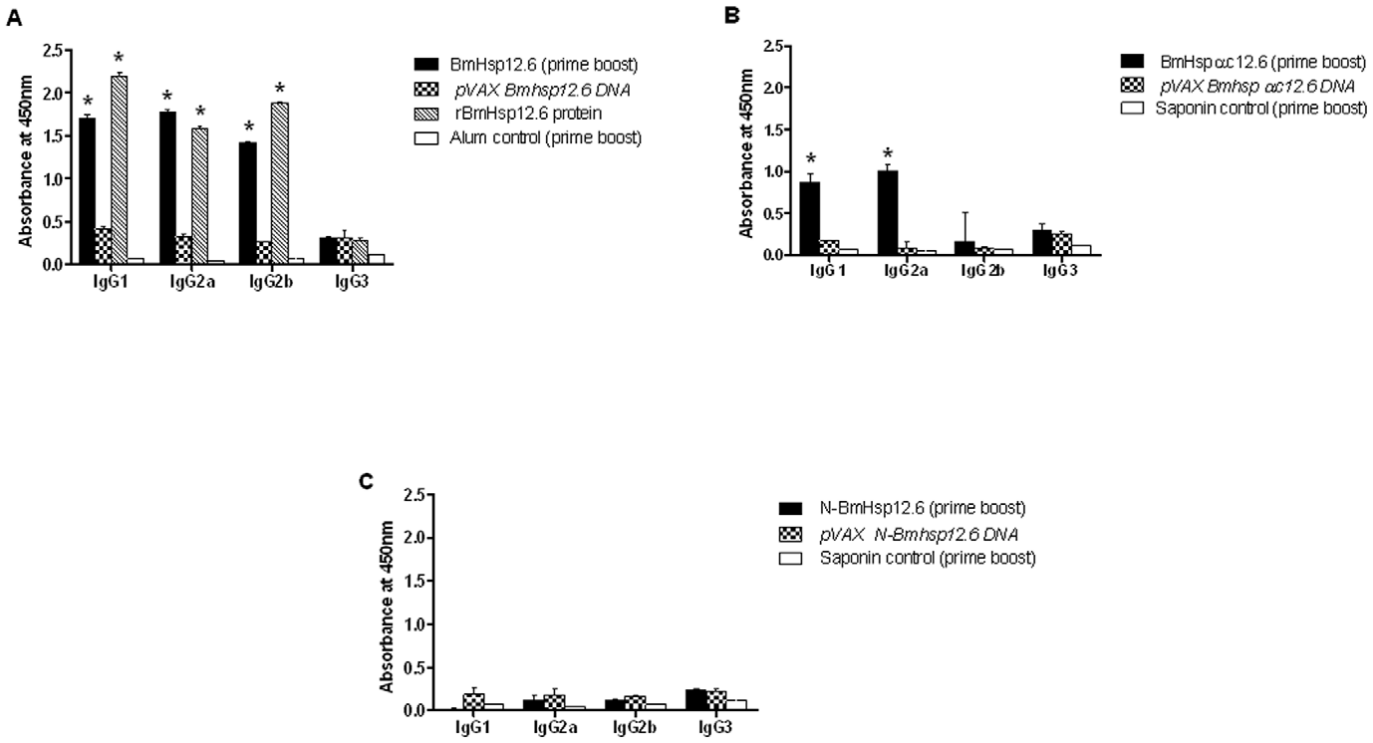
Isotype of anti-BmHsp12.6 IgG antibodies in the sera of mice. Mice were immunized with **A**) BmHsp12.6, **B**) BmHsp12.6αc or **C**) N-BmHsp12.6 using homologous DNA vaccine regimen or a heterologous prime boost approach. Control mice were immunized with vector alone or adjuvant. Isotype specific ELISA was performed as described in the methods section. Bars represent mean O.D ± SD from five mice per group. * Significant (p<0.005) compared to all the other groups.

Analysis of the isotype of anti-BmHsp12.6 IgG antibodies in BmHsp12.6 vaccinated mice showed that predominantly IgG1, IgG2a and IgG2b anti-BmHsp12.6 antibodies were present in the sera of these animals (Figure 9A). IgG1 and IgG2a antibodies were also increased in the sera of rBmHsp12.6αc vaccinated animals (Figure 9B). However, in N-BmHsp12.6 vaccinated animals, there was no significant increase in any IgG isotype of antibodies (Figure 9C).

### Vaccination with BmHsp12.6 and BmHsp12.6αc confers significant protection in mice

Above results showed that significant antibodies are generated in the sera of vaccinated mice. To test if these antibodies can confer protection, we performed an *in vitro* ADCC assay similar to that described for human PBMC. Our results show that serum antibodies from rBmHsp12.6 and BmHsp12.6αc peptide vaccinated mice promoted adherence of peritoneal exudate cells to L3 and participated in larval killing *in vitro* (83% or 81% respectively) compared to sera from control animals (13%), significant p<0.01 (Table 3). However, sera from N-BmHsp12.6 peptide vaccinated mice were less efficient in killing L3 (43%). These findings confirmed that IgG antibodies present in the sera of rBmHsp12.6 and BmHsp12.6αc vaccinated mice are participated in ADCC mediated killing.

**Table 3:** Killing of *B. malayi* L3 in rBmHSP12.6 vaccinated mice was evaluated by *in vitro* (ADCC assay using mouse sera) and *in vivo* (micropore chamber challenge) assays.

	% larval death	
Immunization regimen	*in vitro(ADCC)* a	*in vivo (micropore chamber)* b
N-BmHsp12.6 (prime boost)	43.97±2.71	38±14.8
*pVAX N-BmHsp12.6* DNA	29.76±3.92	31±2.40
BmHsp12.6αc (prime boost)	**81.58±1.68** *	**83±5.72** *
*pVAX BmHsp12.6αc* DNA	47.94±1.34	63±5.50
BmHsp12.6 (prime boost)	**83.02±3.62** *	**72±10.22** *
*pVAX BmHsp12.6 DNA*	43.7±8.12	31±5.23
rBmHsp12.6 protein	55.08±1.15	58±7.76
Control	13±2.35	7±5.2

a ADCC assay was performed by incubating 50 µl of pooled mice sera (n = 5) samples with 2×10^5^ normal peritoneal exudates cells and 10 *B. malayi* L3 at 37°C for 48 hrs. Values represent mean ± SD of three wells.

b *In vivo* micropore chamber assay was performed by surgically implanting 20 *B. malayi* L3 into the peritoneal cavity of each mouse. 48 hrs after implantation, chambers were removed and larval viability and death determined. Values are mean ± SD. N = 5. Data presented is from one of two similar experiments showing comparable results.

* Significant larval death (P<0.01) compared to other mice groups.

Vaccine potential of rBmHsp12.6 and BmHsp12.6αc was further confirmed in the mouse model using a micropore chamber challenge method. Results showed that 72%, 83% and 38% larval death occurred in mice vaccinated with rBmHsp12.6, BmHsp12.6αc or N-BmHsp12.6 protein respectively compared to 7% larval death in controls (Table 3). These findings confirmed that vaccination with both BmHsp12.6αc and rBmHsp12.6 induced significant (P<0.01) protection and were comparable to the ADCC results and human PBMC assays, whereas, mice immunized with N-BmHsp12.6 peptide was not as efficient as the other two proteins. Following DNA vaccination, the larval death were 31%, 63% and 31% in mice vaccinated with *BmHsp12.6*, *BmHsp12.6αc* or *N-BmHsp12.6* DNA respectively suggesting that vaccination with *BmHsp12.6αc* DNA is significantly better than *BmHsp12.6* and *N-BmHsp12.6*. Our results show that prime boost vaccination regimen with BmHsp12.6 and BmHsp12.6αc is highly efficient in conferring vaccine-induced protection against the challenge infection.

### Spleen cells from vaccinated animals showed antigen specific recall response

**Figure 10:**
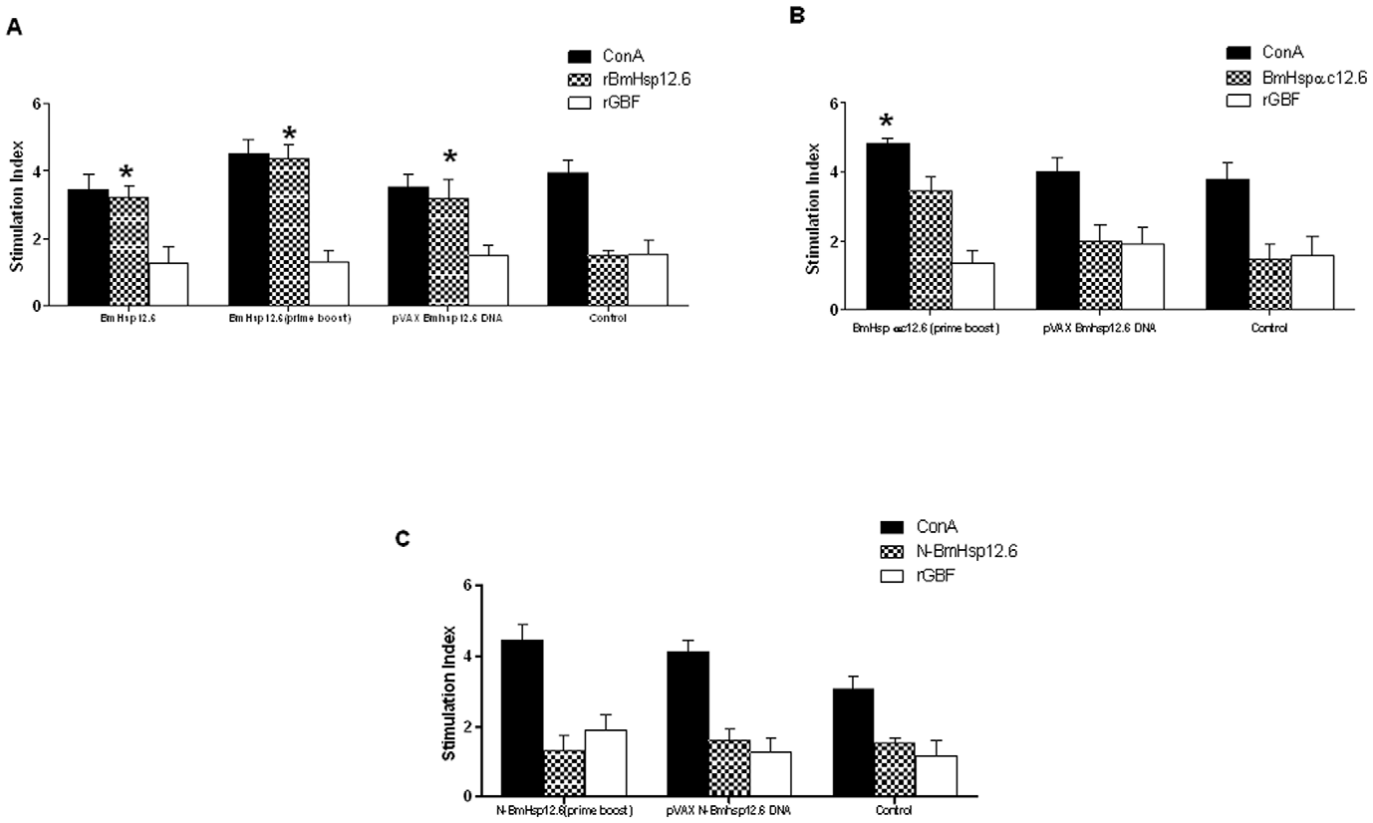
Splenocytes from vaccinated mice proliferated in response to the antigens. Single cell suspension of spleen cells (2×10^5^) from vaccinated and control mice were stimulated with respective peptides or protein for 72 hrs at 37°C. Control wells were either stimulated with Con A (positive control) or left unstimulated (negative control). Mice vaccinated with **A**) BmHsp12.6, **B**) BmHsp12.6αc or **C**) N-BmHsp12.6 using homologous DNA vaccine regimen or a heterologous prime boost approach. A non-specific recombinant protein (rSmGBF) was used as negative control. Data is presented as mean stimulation index (S.I.) of five mice ± S.D. * Significant (p<0.005) S.I. value compared to control cells.

Spleen cells from animals immunized with rBmHsp12.6 (Figure 10A) and BmHsp12.6αc (Figure 10B) and using a prime-boost regimen proliferated significantly (P<0.001) in response to the respective recombinant antigen (SI of 3.43±0.410 and 4.35±0.415). However, spleen cells from N-BmHsp12.6 immunized animals (SI of 1.30±0.434) did not proliferate well in response to N-BmHsp12.6 peptide (Figure 10C). Stimulation index of splenocytes from *pVAX BmHsp12.6* DNA vaccinated group (SI of 3.18±0.565) showed significant proliferation than splenocytes from *pVAX N-BmHsp12.6* DNA (SI of 1.60±0.342) or *pVAX BmHsp12.6αc* DNA (SI of 1.99±0.481) vaccinated animals. Spleen cells from control group of animals failed to proliferate in response to any of the antigens tested (SI 1.46–1.5±0.13–0.15) and was similar to media alone controls.

## Discussion

Experiments described in this manuscript show that *B. malayi* HSP12.6 (BmHsp12.6) is structurally and functionally similar to other members of the class of small heat shock proteins that can act as molecular chaperones. The small heat shock proteins can recognize proteins of non-native structure and prevent them from irreversible intracellular aggregation [36]. Our results showed that BmHsp12.6 can bind to cellular proteins and protect them from heat aggregation or chemical denaturation. These functions may be critical for survival of the parasite in the vertebrate host. Our previous studies reported that BmHsp12.6 can bind to human IL-10R and activate IL-10 mediated effects *in vitro* [10]. In the present study we show that the IL-10R binding region of BmHsp12.6 resides in its N-terminal sequences. Based on this finding it was predicted that BmHsp12.6 has host immunomodulatory function. Thus, neutralizing the effects of BmHsp12.6 might interfere with the establishment of the infective stages of the parasite in the host. Therefore, in this manuscript we evaluated the vaccine potential of BmHsp12.6. Our results show mice vaccinated with rBmHsp12.6 using a heterologous prime boost approach exhibit close to 72% protection against a challenge infection. The α-crystalline domain and C-terminal extension of BmHsp12.6 (BmHsp12.6αc) were found to have the most immunogenic epitopes. This observation correlated with our subsequent findings that majority of the vaccine-induced protection conferred by BmHsp12.6 is associated with BmHsp12.6αc.

Structural analysis of BmHsp12.6 showed that the N-terminal region of BmHsp12.6 has no specific structures. Interestingly, similar intrinsically disordered regions have been described from other small heat shock proteins that can interact with diverse geometries of hydrophobic patches on unfolding proteins [37], [38]. A considerable body of evidence suggests that these intrinsically unstructured regions of proteins play key roles in protein–protein interactions [38], [39]. In fact, the BmHsp12.6αc peptide can only bind with lower intensity to the substrates compared to the intrinsically unstructured N-terminal arm [40]. Thus, the N-terminal arm of BmHsp12.6 appear to be the most favored site for protein-protein interaction and possibly the contact region for potential substrates. At present we do not know if this region is critical for the chaperone function of BmHsp12.6. However, we now know that the N-terminal arm of BmHsp12.6 is involved in the binding of BmHsp12.6 to huIL-10 receptor by *in silico* analysis.

Extensive sequence analysis of the N-terminus region of BmHsp12.6 showed the presence of similar motifs in both BmHsp12.6 and huIL-10. Since protein motifs are signatures of protein families and can often be used as tools for the prediction of protein function, presence of similar motifs in both the IL-10 and BmHsp12.6 suggests that both these proteins may share similar functions. One of these functions appears to be binding to IL-10R. This was further confirmed by the fact that binding of rBmHsp12.6 to huIL-10R interferes with the binding of huIL-10 to huIL-10R suggesting that both proteins may share similar binding sites on huIL-10R. Reinke et al.1998 [41], identified the IL-10/IL-10R combining site by mapping sets of overlapping peptides derived from both binding partners. When the sequence of this binding site was compared to BmHsp12.6 sequences, we found that the N-terminal arm of BmHsp12.6 has identical amino acid sequence to the IL-10R binding peptide of huIL-10. Subsequently, we prepared N-BmHsp12.6 peptide and when tested on MC/9 cells *in vitro* these cells proliferated similar to those induced by rhuIL-10 and full length BmHsp12.6. However, BmHsp12.6αc failed to bind to huIL-10R and failed to stimulate MC/9 cells. These observations strongly suggest that N- terminal sequence of BmHsp12.6 is involved in the binding of BmHsp12.6 to huIL-10R. Similarly the IL-10 like function of BmHsp12.6 also was found to be associated with the N-BmHsp12.6 peptide. Lymphatic filarial parasites are notorious for suppressing host immune responses by inducing an IL-10 response [42]. Thus, it is possible that the N-terminal arm of BmHsp12.6 may have a significant role in the host immune modulatory function. Neutralizing the effects of this protein can have significant host benefit. Further studies using N-BmHsp12.6 peptide are necessary to understand the mechanism of host immunomodulation by this peptide.

Our results support previous findings that small HSPs are excellent vaccine candidates. For example, vaccination of mice with *T. gondii* HSP30 gene, a member of the sHSP family, induced significant protection against challenge infections [43]. Similarly, DNA vaccine encoding hsp60 gene was protective against *M. tuberculosis* in infected mice [44]. Small HSPs also appear to be critical for the survival of the parasite. Over expression of small HSPs in *C. elegans* confer increased lifespan [12], [45]. Similarly, BmHsp12.6 homologue in *Nippostrongulus braziliensis* (Nb-Hsp12.6) was shown to be involved in the survival strategies of the parasitic nematodes in deleterious environmental conditions both outside and inside the host [46]. These findings suggest that BmHsp12.6 may be an important survival protein in the parasite. Therefore neutralizing the effect of BmHsp12.6 can have deleterious effect on the parasite.

We measured the titer and isotype of IgG antibodies against BmHsp12.6 and its peptides in the sera of human subjects. These results showed that putatively immune individuals (EN) carry IgG antibodies against BmHsp12.6 and BmHsp12.6αc subunit but not to N-BmHsp12.6 subunit. This finding showed that BmHsp12.6αc is highly immunogenic in the human host. Epitope analysis of BmHsp12.6 further confirmed this. Depletion of anti-BmHsp12.6 antibodies from the human sera samples significantly reduced the larval killing activity of the sera in ADCC assay suggesting that the antibodies generated against BmHsp12.6 or BmHsp12.6αc peptide may be involved in the protection. Several previous studies show that antibodies play a major role in the protection against lymphatic filariasis [47], [48], [49]. More specifically these antibodies target infective L3, which can be measured *in vitro* using an ADCC assay. In fact ADCC is believed to be one of the principal immunological mechanisms responsible for the clearance of circulating lymphatic filarial parasites in both immune human and animals [4], [5]. Thus, ADCC assay is a good surrogate to measure protection in human. MF and CP individuals also carried antibodies against BmHsp12.6 in their sera. However, these antibodies were mostly IgG2 and IgG4 isotype. Use of these sera in ADCC assay was not effective in killing *B. malayi* L3. Anti-BmHsp12.6 and anti-BmHsp12.6αc peptide antibodies present in the sera of EN subjects were predominantly cryophilic IgG1 and IgG3 antibodies. Other studies have also reported similar antibody responses that are protective in EN subjects [3], [50]–[52].

Our results showed that in addition to the significant antibody responses, the PBMC from EN subjects showed significant recall responses to rBmHsp12.6 antigen *in vitro* suggesting that BmHsp12.6-specific memory cells are present in the circulation of EN subjects. Others have also reported the presence of *B. malayi* or *W. bancrofti*-specific circulating memory cells in EN subjects suggesting that these groups of individuals are immunologically highly responsive against *B. malayi* antigens and parasites [52], [53]. BmHsp12.6 antigen-responding cells predominantly secreted IFN-γ suggesting that a Th1 type immune response may be critical for BmHsp12.6 mediated protection in human. This assumption largely based on our finding that anti-BmHsp12.6 antibodies involved in killing of L3 *in vitro* in the ADCC assay. Similar correlation of IgG1 production with Th1 and inflammatory responses was observed in TB [54]. In fact we found that the anti-BmHsp12.6 antibodies were largely of the IgG1 isotype. Thus, there is a clear correlation between the cellular response and antibody response elicited against BmHsp12.6 antigen in EN subjects. Since the anti-BmHsp12.6 antibodies in the sera of EN subjects were involved in the protection mediated by ADCC, we next wanted to determine the vaccine potential of BmHsp12.6 in a mouse model.

Vaccination of mice with rBmHsp12.6 or BmHsp12.6αc was found to induce significant IgG1 and IgG2a antibody responses. Similarly, immunization of C-terminal domain of *Plasmodium falciparum* HSP70 induced IgG2a antibody response in mice models [55]. Mouse IgG2a antibodies are functionally similar to human IgG1 antibodies [56], and mouse IgG1 antibodies are functionally similar to human IgG4 antibodies. Thus the human correlate of IgG antibody responses against BmHsp12.6 is at least partially similar in the mouse. ADCC assay using sera from vaccinated animals showed that similar to EN subjects, sera samples from mouse vaccinated with rBmHsp12.6 or BmHsp12.6αc was able to kill *B. malayi* L3 *in vitro*. However, sera samples from mice immunized with N-BmHsp12.6 were unable to promote killing of *B. malayi* L3 in ADCC. Lack of immunogenic epitopes in N-BmHsp12.6 and the presence of IL-10R binding region may be responsible for these poor responses. Similar results were observed when mice were challenged with *B. malayi* L3 in the micropore chamber, which is a more physiological milieu for larval growth and survival and yet permits the analysis of host effector mechanisms *in vivo* [57]. In mouse vaccinated with rBmHsp12.6 or BmHsp12.6αc, we observed large number of cells attached to the cuticle of the larvae inside the micropore chamber. There was also significant larval death. Based on these findings we believe that the cells that are attached to the larvae are critical for the effector function as serum was not capable of killing the larvae [5]. Further studies will identify the phenotype and mechanism of effector cell mediated killing in this model.

In our mice studies significant enhancement of protection was achieved against infective L3 when BmHsp12.6 was given as a prime boost vaccination regimen. This is potentially because the DNA vaccine can generate T cells with high affinity and the recombinant protein vaccine can amplify the response [58]. DNA vaccines have been developed against a wide range of pathogens including *S. mansoni* [59], *Mycobacteria* [44], and *Trypanosoma cruzi* [45]. DNA vaccination induces potent cellular responses. However, one of the disadvantages of DNA vaccination is that the levels of humoral immune responses generated is modest. Thus, DNA vaccines may be good as priming agents in combination vaccines. In the present study mice immunized with *BmHsp12.6* DNA vaccine alone gave only 32% protection which supports previous observation that DNA immunization provides only negligible level of protection against parasitic infections [59]. Similarly, vaccination with rBmHsp12.6 protein alone could only confer 58% protection against *B. malayi* infections. However, a combination of *BmHsp12.6* DNA prime and rBmHsp12.6 protein boost vaccination regimen synergistically gave 83% protection against a challenge infection. Similar results have been reported with other heterologous prime/boost vaccination approaches as well [60], [61].

In conclusion, in this study we show that the BmHsp12.6 has significant chaperone activity and its IL-10R binding region was mapped to N-terminal sequence of BmHsp12.6. Small HSPs have been extensively tested as vaccine candidates against several infectious agents and cancer. To demonstrate the vaccine potential of BmHsp12.6, we first showed that putatively immune EN subjects carry anti-BmHsp12.6 IgG1 and IgG3 antibodies in their sera that can participate in the killing of *B. malayi* L3 in an ADCC assay. Subsequent vaccination trial in a mouse model showed that the BmHsp12.6 and BmHsp12.6αc subunit of BmHsp12.6 gave significant protection in mice following a heterologous prime boost regimen. These findings thus show that the BmHsp12.6 can be further developed into a vaccine candidate against *B. malayi*.
